# Suppression of TRPM7 Inhibited Hypoxia-Induced Migration and Invasion of Androgen-Independent Prostate Cancer Cells by Enhancing RACK1-Mediated Degradation of HIF-1*α*

**DOI:** 10.1155/2020/6724810

**Published:** 2020-03-06

**Authors:** Fei Yang, Jiarong Cai, Hailun Zhan, Jie Situ, Wenbiao Li, Yunhua Mao, Yun Luo

**Affiliations:** Department of Urology, The Third Affiliated Hospital of Sun Yat-Sen University, Guangzhou 510630, China

## Abstract

Transient receptor potential melastatin subfamily member 7 (TRPM7) was essential in the growth and metastatic ability of prostate cancer cells. However, the effects and the relevant molecular mechanisms of TRPM7 on metastasis of prostate cancer under hypoxic atmosphere remain unclear. This study investigated the role of TRPM7 in the metastatic ability of androgen-independent prostate cancer cells under hypoxia. First, data mining was carried out to disclose the relationship between the TRPM7 gene level and the survival of prostate cancer patients. Specific siRNAs were used to knockdown target genes. Western blotting and qPCR were employed to determine protein and gene expression, respectively. The gene transcription activity was evaluated by luciferase activity assay of promoter gene. The protein interaction was determined by coimmunoprecipitation. Wound healing and transwell assays were employed to evaluated cell migration and invasion, respectively. Open access database results showed that high expression of TRPM7 was closely related to the poor survival of prostate cancer patients. Hypoxia simultaneously increased TRPM7 expression and induced HIF-1*α* accumulation in androgen-independent prostate cancer cells. Knockdown of TRPM7 significantly promoted HIF-1*α* degradation through the proteasome and inhibited EMT changes in androgen-independent prostate cancer cells under hypoxic condition. Moreover, TRPM7 knockdown increased the phosphorylation of RACK1 and strengthened the interaction between RACK1 and HIF-1*α* but attenuated the binding of HSP90 to HIF-1*α*. Whereas knockdown of RACK1 increased the binding of HSP90 to HIF-1*α*. Furthermore, both TRPM7 and HIF-1*α* knockdown significantly suppressed hypoxia-induced Annexin A1 protein expression, and suppression of HIF-1*α*/Annexin A1 signaling significantly inhibited hypoxia-induced cell migration and invasion of androgen-independent prostate cancer cells. Our findings demonstrate that TRPM7 knockdown promotes HIF-1*α* degradation via an oxygen-independent mechanism involving increased binding of RAKC1 to HIF-1*α*, and TRPM7-HIF-1*α*-Annexin A1 signaling axis plays a crucial role in the EMT, cell migration, and invasion of androgen-independent prostate cancer cells under hypoxic conditions.

## 1. Introduction

Hypoxic environments result from the rapid proliferation of cancer cells leading to insufficient blood supply, which is particularly predominant in prostate cancer. Hypoxia is a typical characteristic of prostate cancer and is a major pathological factor attributing to castration resistance and the metastasis of prostate cancer [1]. When suffering the hypoxic environments, prostate cancer cells regulate a series of gene expression and the corresponding pathways that were essential for cell survival and stress adaptation [2]. Previous researches reported that hypoxia strongly attributed to the poor prognosis and malignancy of prostate cancer [3, 4]. Targeting hypoxia relevant signaling pathways is essential in drug development and would be beneficial for the treatment of prostate cancer.

Hypoxia-inducible factor 1*α* (HIF-1*α*) serving as one of the principal transcription factors is increased by hypoxia and accounts for the regulation of the expression of hypoxia-response genes. Under hypoxia, HIF-1*α* protein expression rapidly accumulates and regulates downstream target gene expression. Whereas under normoxic circumstances, the rapid degradation of HIF-1*α* in the 26S proteasome is mediated by the von Hippel-Lindau (VHL), working together with E3 ubiquitin ligase complex [5]. The degradation of HIF-1*α* is also regulated by an oxygen-independent mechanism involving HIF-1*α* binding to the receptor of activated protein kinase C (RACK1) and Heat Shock Protein 90 (HSP90). RACK1, as a multifunctional anchoring protein, promotes HIF-1*α* degradation. Regarding the binding to HIF-1*α*, RACK1 was showed to compete with HSP90, which is a molecular chaperone and stabilized HIF-1*α*, suggesting that the balance between RACK1 and HSP90 is vital for the degradation of HIF-1*α*. It was demonstrated that HIF-1*α* accumulated in prostate cancer tissues, and HIF-1*α* overexpression was associated with castration resistance, proneness to recurrence, and metastasis in prostate cancer patients [6, 7]. However, the mechanisms involved in HIF-1*α* relevant signaling pathways remain mostly unclear.

Annexin A1 is a glucocorticoid-regulated anti-inflammatory protein, which is also a Ca^2+^ binding protein. Annexin A1 was found to be a direct target of HIF-1*α* which upregulated Annexin A1 expression, while HIF-1*α* knockdown blocked hypoxia-induced Annexin A1 expression [8]. Recently, it was reported that hypoxia stimulus increased Annexin A1 protein expression, and thus to accelerate cell invasion and aggressiveness of prostate cancer cell [9], implying that HIF-1*α*/Annexin A1 signaling played a crucial role in hypoxia-regulated metastasis of prostate cancer.

Transient receptor potential melastatin-like 7 channel (TRPM7) is a nonselective ion channel that is permeable to Ca^2+^, Mg^2+^, and other cations as well. TRPM7 is widely expressed in various types of tissues, especially high levels in heart, bone, and adipose tissue [10]. TRPM7 plays an essential role in numerous physiological processes, including cellular growth, cell death, and development. The deficiency of TRPM7 leads to the inhibition of growth and cell death in B cells and human osteoblastic cells. That also results in the embryonic lethality and thymopoiesis and abnormal acetylcholine release in sympathetic neurons [11]. TRPM7 is also involved in the maintenance of magnesium homeostasis and is critical for the membrane potential and automaticity in myocardial myocytes [12, 13]. Our previous study revealed that TRPM7 expression increased in androgen-independent prostate cancer cells when compared with normal prostate cells [14]. TRPM7 inhibition by Carvacrol suppressed prostate cancer cells' proliferation, migration, and invasion [14]. The subsequent study by other researchers also supported that TRPM7 played an essential role in the aggressiveness of prostate cancer. TRPM7 protein expression was significantly higher in metastatic prostate cancer tissues than that in benign prostatic hyperplasia tissues [15]. Downregulation of TRPM7 inhibited migration and invasion of prostate cancer cells with a mechanism involved in the regulation of epithelial-mesenchymal transition (EMT) [15]. Besides, TRPM7 also mediated transforming growth factor beta- (TGF *β*-) induced EMT in prostate cancer [16]. TRPM7 overexpression was induced by hypoxia-ischemia in the rodent brain and mediated the corresponding brain injury, serving as a potential drug target for stroke [17]. However, it remains largely unknown about the role of TRPM7 in hypoxic signaling in prostate cancer. Here, we proposed a hypothesis that TRPM7 might respond to hypoxia to regulate the degradation of HIF-1*α*, and thus to control its downstream target, Annexin A1, and subsequently to affect the hypoxia-induced aggressive ability of androgen-independent prostate cancer cells.

## 2. Materials and Methods

### 2.1. Data Mining in Human Protein Atlas Database

The relationship between TRPM7 gene expression and the prognosis of prostate cancer patients was mining from the Human Protein Atlas (HPA) database (http://www.proteinatlas.org/), as described previously [18]. The survival data were extracted from this database, and the survival analysis was plotted using the log-rank test.

### 2.2. Cell Culture and Cell Treatment

DU145 (HTB-81) and PC3 (CRL1435) as androgen-independent prostate cancer cell lines were purchased from the American Type Culture Collection (ATCC, USA). Both cell lines were grown in DMEM containing 10% fetal bovine serum, penicillin (100 U/mL), and streptomycin (100 ng/mL) at 37°C with 5% CO_2_. All the cell culture-related materials were purchased from Gibco Company, USA, unless mentioned otherwise. For hypoxia experiments, cells were grown in hypoxia incubator (Thermo Scientific, USA) in 1% oxygen for indicated time point. 20% oxygen was used as the normoxic control.

### 2.3. siRNA and Gene Knockdown

Negative control siRNA and siRNA specific to TRPM7, HIF-1*α*, RACK1, and Annexin A1 were synthesized by Sangon Biotech (Shanghai, China). The negative control siRNA and TRPM7-specific-siRNA used the sequences as described in our previous study [14]. The sequences of HIF-1*α*-specific siRNA was 5′-CTGATGACCAGCAACTTGA-3′ [19]. Annexin A1-specific siRNA was 5′-ACUCCAGCGCAAUUUGAUGTT-3′ [20]. RACK1-specific siRNA was 5′-AAGCTGAAGACCAACCACA-3′. siRNA transfection was carried out using Lipofectamine RNAiMAX transfection reagent (Thermo Fisher Scientific). Cells were seeded into 6-well plates, and the siRNA transfections were performed following the product's instruction. After 72 h of transfection, cells were exposed to hypoxic or normoxic conditions, and the protein expressions and functional experiments were performed.

### 2.4. Western Blot (WB) and Immunoprecipitation

Cells were washed three times with PBS buffer and then lysed using RIPA buffer (Beyotime, China) on ice for 30 min. The bicinchoninic acid (BCA) kit (Beyotime, China) was used to determine the total protein concentration in samples. Then, equivalent protein of each group was separated on 8–12% SDS-PAGE gels and transferred to PVDF membrane (Millipore, USA). After blocking with 5% BSA in TBS with 0.1% tween-20, transferred membranes were incubated with primary antibodies overnight. The diluted information of primary antibody were as follows: anti-TRPM7 (1 : 1000, Sigma, USA; Cat#: SAB5200032), anti-E-Cadherin (E-Cad, 1 : 1000, Cell Signaling Technology, USA; Cat#: 14472), anti-N-Cadherin (N-Cad, 1 : 1000, Cell Signaling Technology, USA; Cat#: 13116), anti-vimentin (1 : 1000, Cell Signaling Technology, USA), anti-HIF-1*α* (1 : 1000, Cell Signaling Technology, USA; Cat#: 5741), anti-Annexin A1 (1 : 1000, Cell Signaling Technology, USA; Cat#: 32934), and anti-*β*-actin (1 : 1000, Cell Signaling Technology, USA; Cat#: 4970). After washed with TBST three times, membranes were incubated with corresponding horseradish peroxidase-conjugated secondary antibodies (1 : 5000, Cell Signaling Technology, USA). The blots were detected by a chemiluminescence reagent system (Beyotime, China).

Coimmunoprecipitations between HIF-1*α* and RACK1/HSP90 followed the protocol from Cell signaling company. In brief, lysates were incubated with ab-HIF-1*α* (1 : 50, Cell Signaling Technology, USA; Cat#: 36169) or Rabbit mAb IgG (Cell Signaling Technology, USA; Cat#: 3900) using as negative control overnight, followed by addition of protein A-agarose beads (Invitrogen). Beads were washed with lysis buffer and proceeded to WB assay as the above description. RACK1 antibody (1 : 1000, Cat#: 5432) and HSP90 (1 : 1000, Cat#: 4877) antibody were purchased from Cell Signaling Technology, USA.

### 2.5. Real-Time Quantitative PCR (qPCR)

After the cells completed the indicated treatments, total RNA of each treatment group was extracted using TRIzol reagent (Invitrogen) and reversely transcribed into cDNA using a cDNA synthesis kit (Thermo Fisher Scientific) according to the product's instruction. Quantitative PCR was carried out using a SYBR Green Master Mix (Bio-Rad) in ABI 7700 system. The primer sequences for HIF-1*α* and *β*-actin were as follows: HIF-1*α*, forward, 5′-TATGAGCCAGAAGAACTTTTAGGC-3′ and reverse, 5′-CACCTCTTTTGGCAAGCATCCTG-3′; and *β*-actin, forward, 5′-AAGGATTCCTATGTCGGC-3′ and reverse, 5′-CTTCATGATGGAGTTGAAGGT-3′. The reaction parameters were carried out as the following conditions: 95°C for 30 sec, 95°C for 15 sec, and 60°C for 30 sec with 40 cycles. Relative gene expression values were calculated by the 2^−*Δ*CT^ method. The expression of HIF-1*α* was normalized by using the expression of *β*-actin. Data were presented as the percentage change of the control group.

### 2.6. Luciferase Activity Assay

The RACK1 promoter was subcloned into the pGL3 basic vector. After cells were transfected with control siRNA or TRPM7 siRNA for 24 h, cells were transfected with one of the constructed vectors or the pGL3 basic vector with Lipofectamine 3000 (Invitrogen) following the manufacturer's instruction. 24 h after transfection, cells were exposed to hypoxic or normoxic conditions for an additional 24 h. Then cells were lysed, and the measurement of luciferase activity was performed using the luciferase assay system (Promega). The luciferase activity was normalized by the protein concentration.

### 2.7. RACK1 Overexpression Plasmid Construction and Transfection

The whole cDNA sequences of human Rack1 (NM_006098.4) were amplified and subcloned into plasmid pcDNA3.1myc/His/Neo. The inserted region was between Hind III and Not I. The prostate cancer cells were transfected with pcDNA3.1-Rack1 (Rack1) or pcDNA3.1 vector (vector) using the Lipofectamine 3000 according to the manufacturer's instructions (Invitrogen) for 48 h.

### 2.8. Wound Healing Assay

Wound healing assay was carried out as described previously [14]. Briefly, cells were grown in 6-well plates and starved in a serum-free medium for 24 h. The wound gaps were created by using a 200 *μ*L pipette tip. The gaps were marked using a black color marker pen to guide the image capture of wound gaps. The closure of the wound gap was monitored by photographing using a phase-contrast Olympus microscope at indicated time point. The gap area was measured by ImageJ software. The wound closure was calculated using the formula as follows: wound closure (%) = (G_0_ − G_t_)/G_0_∗100 (where G_t_ was the gap area after treatment, and G_0_ is the first gap area when the wound was induced).

### 2.9. Invasion Assay

Transwell assay was carried out as described in our previous study [14]. Briefly, cells were starved in a serum-free medium for 24 h and then seeded at a density of 2.5 × 10^4^ cells/mL in FBS-free DMEM in the upper chamber. The lower chamber was added 600 *μ*L medium with 10% FBS. After incubating for 24 h in the cell culture incubator, cells in the upper chamber were using a cotton swab. Invaded cells in the bottom surface were fixed and stained with crystal violet (0.1%, Sigma, USA). After washed with water, images of the invaded cells were photographed. Then, crystal violet stained cells were lysed with 10% acetic acid, and the absorbance value of the lysates was read at 490 nm using a microplate reader (Synergy H1, BioTek, USA). The OD values were used to quantitate the invaded cells.

### 2.10. Statistical Analysis

Data are presented as mean ± SD. The difference between the two groups was analyzed using the two-way unpaired Student's *t*-test. One-way ANOVA with subsequent Tukey-Kramer post hoc test was used for multiple comparisons when more than two groups. The value of *p* < 0.05 was considered statistically significant.

## 3. Results

### 3.1. High Level of TRPM7 Gene Was Closely Associated with Poor Prognosis of Prostate Cancer

**Figure 1:**
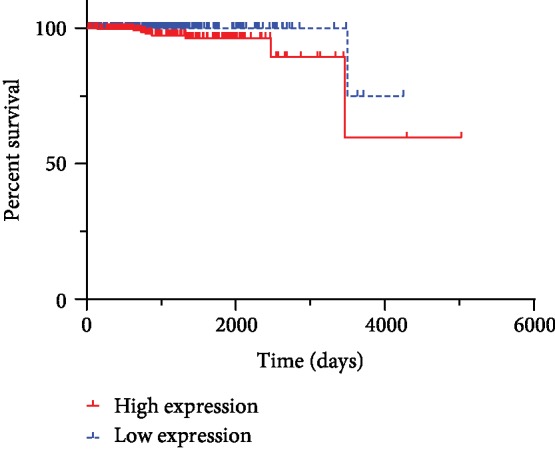
The prognosis of the prostate cancer patient with different levels of TRPM7 gene expression. The survival analysis result was from the HPA database using the best expression cutoff. The log-rank Kaplan-Meier survival analysis result was shown (*p* = 0.016).

Human Protein Atlas (HPA) is open access to the researcher to analyze the relationship between gene level and survival of cancer patients. The relationship between TRPM7 expression and survival of prostate cancer patients is unclear. By utilizing the HPA database, the TRPM7 gene levels and the survival of prostate cancer patients were obtained. Patients were divided into low_TRPM7 and high_TRPM7 groups. If the cutoff was used with the medium expression of TRPM7 gene, there was no significant difference between high and low TRPM7 gene expression groups (*p* = 0.095). However, if the best expression cutoff at 3.35 fragments per kilobase of transcript per million mapped reads (FPKM) was used. As shown in Figure 1, the *p* value of the Kaplan-Meier survival analysis result from HPA was 0.016, suggesting that the high expression of TRPM7 gene in prostate cancer patients was closely associated with poor survival of prostate cancer.

### 3.2. Hypoxia-Induced Increase of TRPM7 Protein Expression-Mediated EMT Change of Androgen-Independent Prostate Cancer Cells

**Figure 2:**
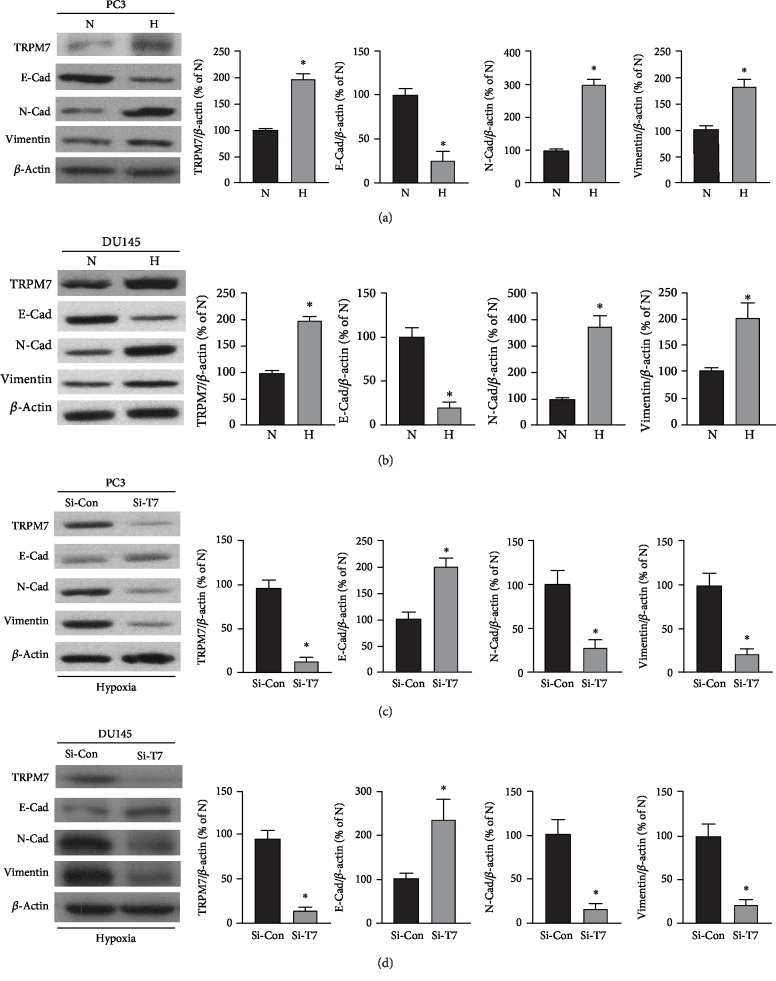
The effects of TRPM7 on hypoxia-induced EMT in androgen-independent prostate cancer cells. (a, b) PC3 (a) and DU145 cells (b) were grown in either hypoxic condition (H) or normoxic condition (N) for 24 h. TRPM7 protein and EMT markers (E-Cad, N-Cad, and vimentin) were determined using western blotting. The representative images and the densitometry analyzed results were shown. ∗ versus normoxia group, *p* < 0.05, *n* = 4. (c, d) PC3 (c) and DU145 cells (d) were carried out to knockdown TRPM7 by TRPM7 siRNA (Si-T7), and negative siRNA (Si-Con) was used as control. Cells were suffered hypoxia for 24 h. TRPM7 protein and EMT markers (E-Cad, N-Cad, and vimentin) were determined using western blotting. The representative images and the densitometry analyzed results were shown. ∗ versus negative control siRNA group, *p* < 0.05, *n* = 5.

Exposure to hypoxic conditions is a common characteristic over the growing periods of advanced solid cancers, including prostate cancer, which shows to promote invasive behavior of prostate cancer cells [1]. In neurons, TRPM7 responded to hypoxia and regulated cell death [21]. It remains unclear if TRPM7 expression was regulated by hypoxia in prostate cancer cells. HIF-1*α* protein expression was determined by western blotting to validate the hypoxic condition exposed in androgen-independent prostate cancer cells. As shown in [Supplementary-material supplementary-material-1], HIF-1*α* protein expression significantly increased in both PC3 and DU145 cells. As shown in Figures 2(a) and 2(b), western blotting results showed that the androgen-independent prostate cancer cells, PC3 and DU145 subjected to hypoxia exposure, significantly increased TRPM7 protein expression as compared with normoxic conditions (*p* < 0.05, *n* = 4). In the meantime, the results showed that hypoxia-induced EMT in androgen-independent prostate cancer cells along with increased TRPM7 expression, showing as hypoxia-induced significant decrease of E-cadherin (E-Cad) while an induced increase of expression of vimentin and N-cadherin (N-Cad).

Next, TRPM7 protein expression was downregulated by using siRNA to determine if TRPM-mediated hypoxia-induced EMT change in androgen-independent prostate cancer cells. As shown in Figures 2(c) and 2(d), under hypoxic conditions, TRPM7 knockdown by siRNA in both PC3 and DU145 significantly suppressed the alteration of EMT marker protein expression induced by hypoxia. TRPM7 knockdown significantly increased E-Cad protein expression while it significantly reduced N-Cad as well as vimentin protein expression (*p* < 0.05, *n* = 5).

### 3.3. TRPM7 Knockdown Suppressed HIF-1*α* Protein Expression, and HIF-1*α* Regulated the EMT in Hypoxia-Insulted Androgen-Independent Prostate Cancer Cells

**Figure 3:**
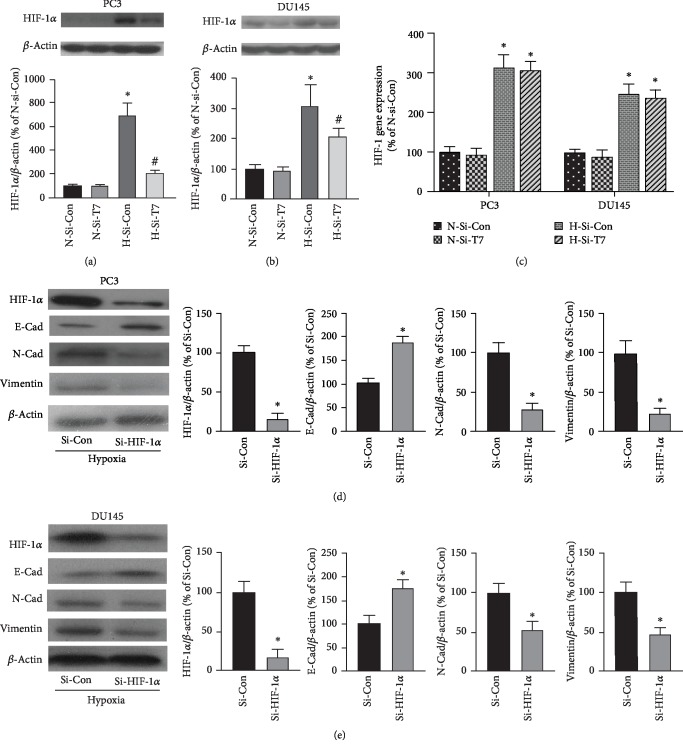
Knockdown of TRPM7 promoted the degradation of HIF-1*α* protein under hypoxia in androgen-independent prostate cancer cells. (a, b) PC3 (a) and DU145 cells (b) were transfected with TRPM7-siRNA (Si-T7) or negative control siRNA (Si-Con) for 72 h; then they were exposed to hypoxia (H) or normoxia (N) for 24 h. The HIF-1*α* protein expression was determined by using western blot. The representative blot images were shown. ∗ versus normoxia group with the transfection of Si-Con (N-Si-Con) group, # versus hypoxia group with the transfection of si-Con (H-Si-Con) group, *p* < 0.05, *n* = 5. (c) The gene expression of HIF-1*α* in prostate cancer cells transfected with control siRNA or Si-T7 under either normoxic or hypoxic conditions as described above. Quantitative PCR was carried out to determine the HIF-1*α* gene expression. The results were presented as the percentage of N-Si-Con. ∗ versus N-Si-Con, *p* < 0.05, *n* = 6. (d, e) PC3 (d) and DU145 cells (e) were transfected with HIF-1*α*-siRNA (Si-HIF-1*α*) or negative control siRNA (Si-Con) for 72 h, and then the cells were exposed to hypoxia. HIF-1*α*, E-Cad, N-Cad, and vimentin protein expressions were determined by western blot. ∗ versus Si-Con group, *p* < 0.05, *n* = 5.

HIF-1*α* accumulation is associated with hypoxia-induced EMT in prostate cancer cells [22]. Next, we determined whether the level of HIF-1*α* protein expression was affected by TRPM7. As shown in Figures 3(a) and 3(b), HIF-1*α* protein expression was significantly increased in both PC3 and DU145 cells when they were insulted by hypoxia for 24 h (*p* < 0.05, *n* = 5). TRPM7 knockdown (H-Si-T7) significantly reduced HIF-1*α* protein expression when compared with the hypoxia group without TRPM7 intervention (H-Si-Con, *p* < 0.05, *n* = 5). Next, we employed real-time qPCR to determine the gene expression of HIF-1*α*. The gene expression of HIF-1*α* was normalized using the gene expression of *β*-actin and was represented as the percentage of control (N-Si-Con). However, TRPM7 knockdown did not significantly change the gene expression of HIF-1*α* induced by hypoxia (Figure 3(c)). These results indicated that TRPM7 knockdown promoted the degradation of HIF-1*α* of androgen-independent prostate cancer cells in hypoxic conditions. HIF-1*α* is involved in EMT of prostate cancer cells [22]. Our western blot results showed that knockdown of HIF-1*α* inhibited EMT change of androgen-independent prostate cancer cells induced by hypoxia in both PC3 and DU145 cells. As shown in Figures 3(d) and 3(e), HIF-1*α*-targeted siRNA significantly reduced HIF-1*α* protein expression induced by hypoxia. In the meantime, E-Cad protein expression significantly increased, whereas N-Cad and vimentin protein expression was significantly reduced by HIF-1*α* knockdown compared with the negative siRNA control group (*p* < 0.05, *n* = 5).

### 3.4. TRPM7 Knockdown Increased the Phosphorylation of RACK1 and Strengthened the Binding of RACK1 to HIF-1*α*

**Figure 4:**
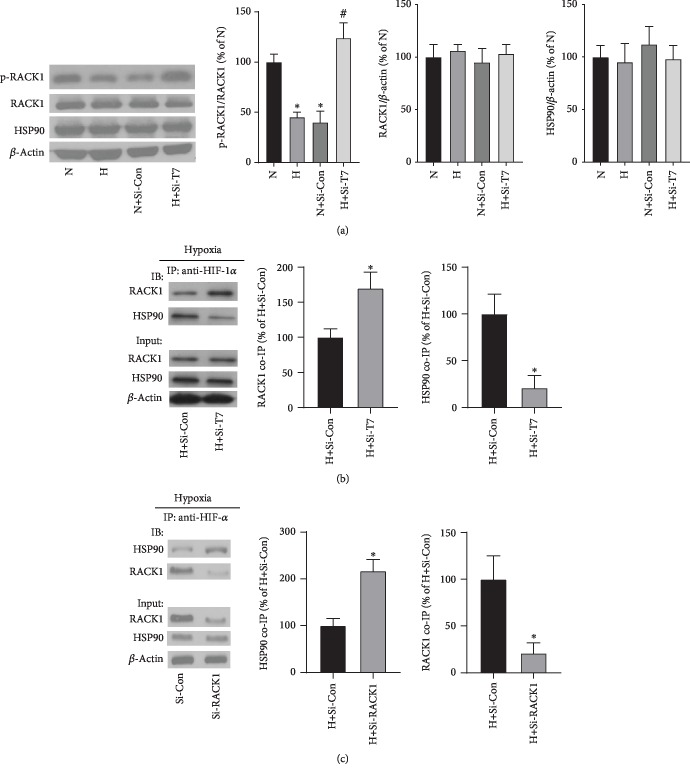
TRPM7 knockdown restored the phosphorylation of RACK1 (p-RACK1), while enhanced the interaction between RACK1 and HIF-1*α* in PC3 cells. (a) Western blotting results showed the protein expression of p-RACK1, RACK1, and HSP90 in PC3 prostate cancer cells under normoxic (N), hypoxic conditions (H), and hypoxia plus siRNA control (H + Si − Con) or siRNA-TRPM7 (H + Si − T7) for 24 h. ∗, # versus N and H + Si − Con, respectively, *p* < 0.05, *n* = 6. (b) Co-IP of HIF-1*α* with RACK1 and HSP90 after TRPM7 knockdown in PC3 prostate cancer cells under hypoxic condition. ∗ versus H + Si − Con, *p* < 0.05, *n* = 4. (c) Co-IP of HIF-1*α* with RACK1 and HSP90 after RACK1 knockdown in PC3 prostate cancer cells under hypoxic condition. ∗ versus H + Si − Con, *p* < 0.05, *n* = 4.

HIF-1*α* proteasomal degradation is regulated by VHL-mediated oxygen-dependent pathway as well as an oxygen-independent pathway that is mediated by HIF-1*α* binding proteins, RACK1, and HSP90 [23]. As shown in Figure 4(a), hypoxia and TRPM7 knockdown did not significantly change the total protein expression of RACK1 and HSP90 protein expression in PC3 cells. Whereas compared with the normoxic conditions (N), hypoxia (H) significantly reduced the phosphorylation of RACK1, which activates RACK1 dimerization [24]. The results showed that TRPM7 knockdown significantly increased the phosphorylation of RACK1. Next, the co-IP results showed that TRPM7 knockdown significantly increased the interaction of RACK1 with HIF-1*α* but reduced the binding of HSP90 to HIF-1*α* in PC3 cells (Figure 4(b)). There was no band observed in the IgG negative control ([Supplementary-material supplementary-material-1]). Moreover, our results showed that RACK1 knockdown by siRNA RACK1 increased the binding of HSP90 with HIF-1*α* in PC3 cells, which was consistent with the previous study indicating that RACK1 competes with HSP90 binding to HIF-1*α* [23] (Figure 4(c)). Besides, the effects of TRPM7 knockdown on the phosphorylation of RACK1 and the interaction between RACK1 and HIF-1*α* in DU145 cells were consistent with the results in PC3 cells ([Supplementary-material supplementary-material-1]).

### 3.5. TRPM7 Knockdown Promoted HIF-1*α* Degradation in the Proteasome

**Figure 5:**
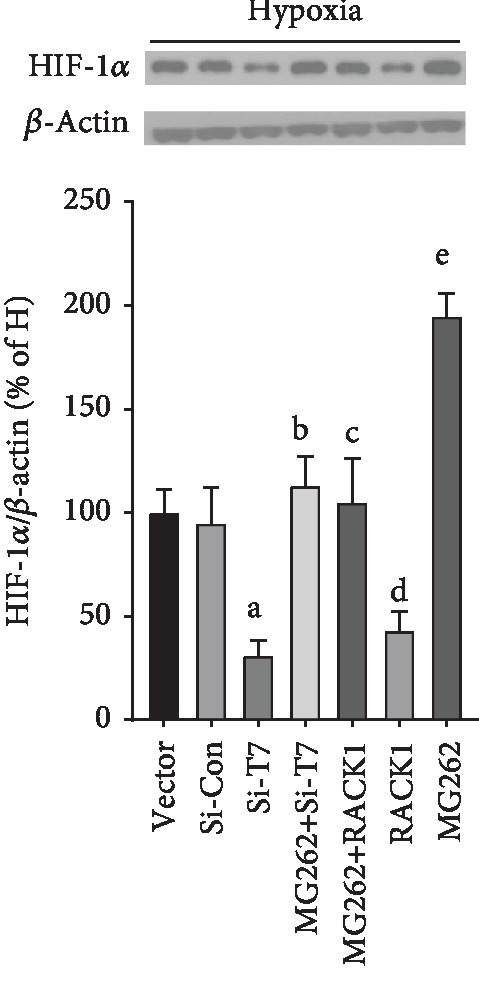
TRPM7 and RACK1 regulated HIF-1*α* degradation via the proteasome under hypoxia. PC3 prostate cancer cells with or without knockdown of TRPM7 (Si-T7) or overexpression of RACK1 (RACK1 group) were incubated with MG262 (1 *μ*M) for 6 h. HIF-1*α* protein expression was determined using western blot. ^a^Versus Si-Con group, ^b^versus Si-T7 group, ^c^versus RACK1 group, ^d^versus vector group, and ^e^versus Si-Con group, *p* < 0.05, *n* = 4.

Both oxygen-dependent and oxygen-independent degradation of HIF-1*α* occurred in the proteasome. Next, we used a proteasome inhibitor, MG262, in combination with siRNA TRPM7 or RACK1 overexpression to determine if TRPM7 knockdown promoted HIF-1*α* degradation through the proteasome. As shown in Figure 5, MG262 significantly attenuated the decrease of HIF-1*α* protein levels induced either by TRPM7 knockdown or RACK1 overexpression (*p* < 0.05, *n* = 4). These results suggest that proteasome is required for the degradation of HIF-1*α* by TRPM7 knockdown and RACK1 overexpression.

### 3.6. Both TRPM7- and HIF-1*α*-Regulated Annexin A1 Expression in Hypoxia-Insulted Androgen-Independent Prostate Cancer Cells

**Figure 6:**
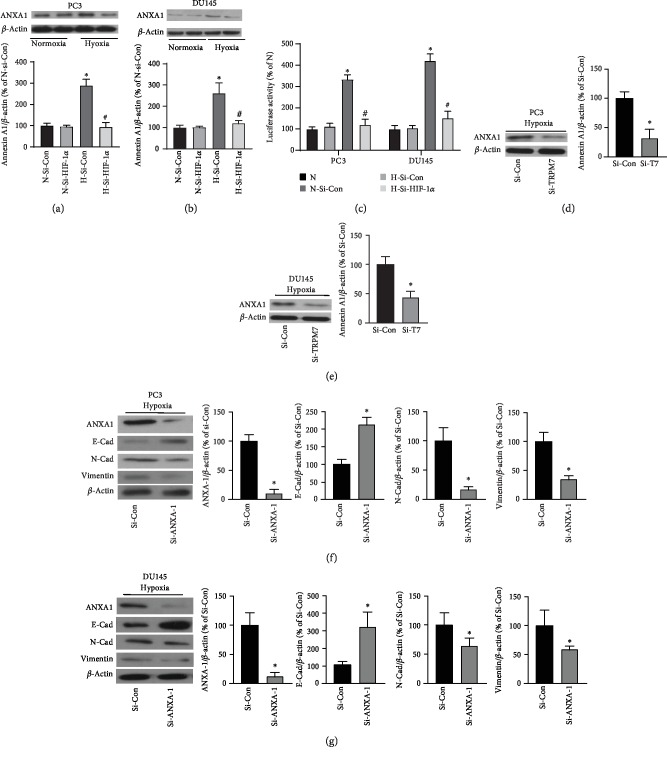
Annexin A1 expression increased by hypoxia was regulated by HIF-1*α* and the effects of knockdown of Annexin A1 on EMT in androgen-independent prostate cancer cells. PC3 (a) and DU145 cells (b) were transfected with HIF-1*α*-siRNA(Si-HIF-1*α*) or negative control siRNA (Si-Con) for 72 h, and then they were exposed to hypoxia (H) or normoxia (N) for 24 h. Annexin A1 protein expression was determined by western blot. ∗ versus normoxia plus Si-Con (N-Si-Con) group, # versus hypoxia plus si-Con (H-Si-Con) group, *p* < 0.05, *n* = 5. (c) The transcription activities of Annexin 1 promoter reporter were measured by the luciferase assay under normoxic or hypoxic conditions with or without HIF-1*α* knockdown in PC3 and DU145 cells. ∗ versus N group, # versus H-Si-Con group, *p* < 0.05, *n* = 6. PC3 (d) and DU145 cells (e) were transfected with TRPM7-siRNA (Si-T7) or negative control siRNA (Si-Con) for 72 h, and then they were exposed to hypoxia for 24 h. Annexin A1 protein expression was determined by western blot. ∗ versus Si-Con group, *p* < 0.05, *n* = 4. PC3 (f) and DU145 cells (g) were transfected with Annexin A1-siRNA (Si-ANXA1) or negative control siRNA (Si-Con) for 72 h, and then they were exposed to hypoxia (H) or normoxia (N) for 24 h. Annexin A1 (ANXA1), E-Cad, N-Cad, and vimentin protein expressions were determined by western blot. ∗ versus si-Con group, *p* < 0.05, *n* = 5.

The downstream target of HIF-1*α* contributing to the hypoxic adaptation of prostate cancer cells remains unclear. The previous study revealed that Annexin A1 was upregulated by HIF-1*α* overexpression induced by hypoxia and played an essential role in prostate cancer [8]. Thus, we determined if Annexin A1 expression was affected by HIF-1*α* in androgen-independent prostate cancer cells. As shown in Figures 6(a) and 6(b), hypoxia significantly increased Annexin A1 protein expression in both PC3 and DU145 prostate cancer cells when compared with prostate cancer cells exposed to normoxic conditions. Whereas HIF-1*α* knockdown significantly reduced Annexin A1 protein expression induced by hypoxia when compared with cells treated with negative control siRNA (*p* < 0.05, *n* = 5). Moreover, the Annexin A1 promoter activity was determined. As shown in Figure 6(c), HIF-1*α* knockdown significantly reduced Annexin A1 promoter activity induced by hypoxia, suggesting that HIF-1*α* regulated Annexin A1 gene transcription. Moreover, TRPM7 knockdown significantly attenuated Annexin A1 protein expression induced by hypoxia (Figures 6(d) and 6(e)).

Next, we determined the effects of Annexin A1 on hypoxia-induced EMT and invasion of androgen-independent prostate cancer cells. As shown in Figures 6(f) and 6(g), Annexin A1 knockdown by siRNA significantly suppressed EMT change of prostate cancer cells induced by hypoxia which was showing as decreased expression of the epithelial marker E-cad and increasing expression of the mesenchymal marker N-Cad and vimentin. This suggested that Annexin A1 downregulation mediated the suppression of EMT induced by HIF-1*α* knockdown in androgen-independent prostate cancer cells.

### 3.7. Suppression of HIF-1*α*/Annexin A1 Signaling Inhibited the Migration and Invasion of Androgen-Independent Prostate Cancer Cells

**Figure 7:**
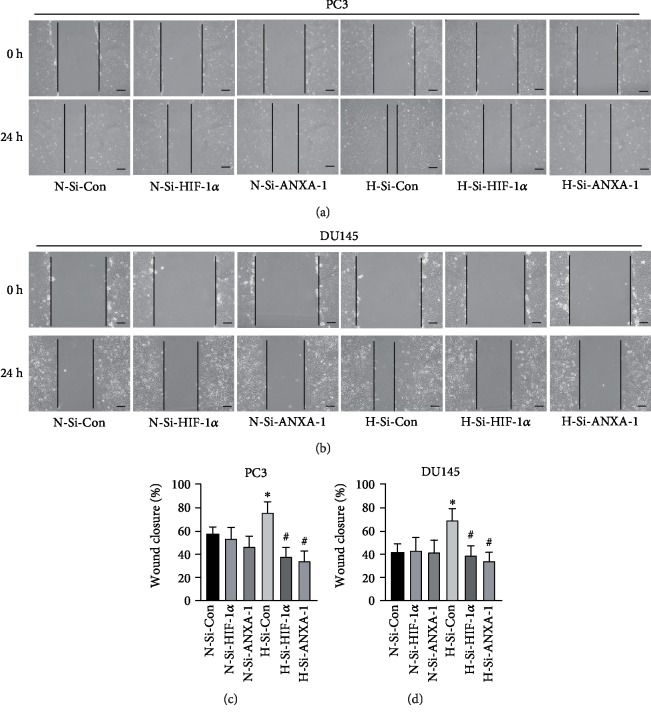
Suppression of HIF-1*α*/Annexin A1 signaling reduced prostate cancer cell migration induced by hypoxia. PC3 (a) and DU145 cells (b) were transfected with HIF-1*α*-siRNA (Si-HIF-1*α*), Annexin A1-siRNA (Si-ANXA1), or negative control siRNA (Si-Con) for 72 h. The wound gaps were created, and the images were photographed (0 h). Then, they were exposed to hypoxia (H) or normoxia (N) for 24 h, and images were photographed (24 h). The wound closure of PC3 (c) and DU145 cells (d) was quantitated. ∗ versus normoxia plus Si-Con (N-Si-Con) group, # versus hypoxia plus si-Con (H-Si-Con) group, *p* < 0.05, *n* = 6.

Finally, the effects of HIF-1*α*/Annexin A1 signaling on hypoxia-induced cell migration and invasion were determined. Wound healing assay was used to evaluate cell migration. As shown in Figures 7(a) and 7(c), the wound closure of PC3 cells which were exposed to hypoxia was significantly faster than that of the control group with normal oxygen exposure (*p* < 0.05, *n* = 6). Under hypoxic condition, both knockdown of HIF-1*α* and Annexin A1 by siRNA significantly reduced wound closure of PC3 cells when compared with PC3 treated with negative control siRNA (*p* < 0.05, *n* = 6). The effects of HIF-1*α* and Annexin A1 knockdown on wound closure of DU145 cells were consistent with that of PC3 cells (Figures 7(b) and 7(d)).

**Figure 8:**
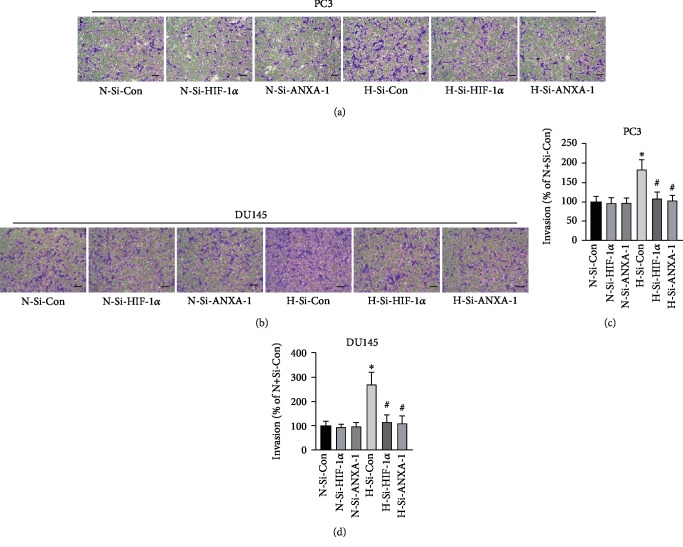
Suppression of HIF-1*α*/Annexin A1 signaling inhibited the invasion of androgen-independent prostate cancer cells. PC3 (a, c) and DU145 cells (b, d) were transfected with HIF-1*α* -siRNA (Si-HIF-1*α*), Annexin A1-siRNA (Si-ANXA1), or negative control siRNA (Si-Con) for 72 h. Then, the transwell assays were carried out. Images of invaded cells with crystal violet staining were photographed. The number of invaded cells was quantitated using a plate reader. ∗ versus normoxia plus Si-Con (N-Si-Con) group, # versus hypoxia plus si-Con (H-Si-Con) group, *p* < 0.05, *n* = 6.

Transwell assay was employed to determine cell invasion. As shown in Figures 8(a)–8(d), hypoxia significantly increased the invasion of PC3 and DU145 cells when compared with cells exposed to normal oxygen (*p* < 0.05, *n* = 6). Whereas either HIF-1*α* or Annexin A1 knockdown significantly reduced the invasion of PC3 and DU145 cells induced by hypoxia when compared cells treated with negative control siRNA in hypoxia condition (*p* < 0.05, *n* = 6).

## 4. Discussion

This study demonstrated that hypoxia increased TRPM7 expression and simultaneously induced HIF-1*α* accumulation as well as EMT in androgen-independent prostate cancer cells. Suppression of TRPM7 promoted HIF-1*α* degradation in proteasome via enhancing the phosphorylation of RACK1 and subsequently increasing the binding of RACK1 to HIF-1*α*. Moreover, knockdown of RACK1 resulted in increased binding of HSP90 to HIF-1*α* to stabilize HIF-1*α* protein. Furthermore, our results showed that both knockdown of HIF-1*α* and TPRM7 significantly reduced Annexin A1 protein expression induced by hypoxia. Our result demonstrated that Annexin A1 served as a downstream target for TRPM7-HIF-1*α* signaling, contributing to the regulation of EMT change, migration, and invasion induced by hypoxia in androgen-independent prostate cancer cells.

TRPM7 expression increased in prostate cancer cells regulating Ca^2+^ and Mg^2+^ influx, and thus leading to an increase of cell proliferation [25]. It was further revealed that increased Ca^2+^ in culture medium (from 1.0 to 2.5 mM) significantly promoted prostate cell proliferation, which was involved in the stromal interaction molecule 1 (STIM1) overexpression [26]. When TRPM7 was activated by cholesterol, cell proliferation and migration of prostate cancer cells were increased [27]. In contrast, blockage of TRPM7 increased TNF-related apoptosis-inducing ligand- (TRAIL-) induced apoptosis of prostate cancer cells [28]. Our previous study also found a higher level of TRPM7 expression in androgen-independent prostate cancer cells comparing with normal prostate cells. At the same time, blockage of TRPM7 by Carvacrol significantly reduced cell proliferation, migration, and invasion [14]. Therefore, current experimental research findings strongly indicated that TRPM7 played an oncogenic role in prostate cancer and likely served as a potential therapeutic target for cancer treatment [29]. It remains unclear in terms of the role of TRPM7 in the prognosis of prostate cancer patients. The previous study demonstrated that TRPM7 protein expression was increased in metastatic prostate cancer tissues when compared with benign prostatic hyperplasia tissues [15]. Here, we presented that the result mining from the HPA database showed that when the best expression cutoff was used, a high level of TRPM7 gene expression in prostate cancer displayed poorer prognosis when compared with patients with low TRPM7 gene expression. It suggests that the high expression of TRPM7 is closely associated with the progression of prostate cancer. However, according to the instruction of HPA database, TRPM7 is still unable to consider as a prognostic gene of prostate cancer. The HPA database might include a heterogeneous group of patients with either castration-sensitive prostate cancer or castration-resistant prostate cancer. Most prostate cancers would progress to castration-resistant prostate cancer with poor prognosis following androgen deprivation treatment [30]. PC3 and DU145 cells are highly metastatic potential and androgen independence, thereby using in the study of aggressive metastatic castrate-resistant prostate cancer. This study, as well as previous researches as described above, uses androgen-independent prostate cancer cell lines, which might cause the discrepancy of the effects of TRPM7 in prostate cancer between the HPA database and these in vitro studies. Further clinical research with a larger sample size is required to reveal the clinical value of TRPM7 in prostate cancer.

Hypoxia is a common characteristic of prostate cancer and attributes cancer progression and poor prognosis [31]. Moreover, hypoxia also protected against androgen deprivation and was associated with the resistance of chemotherapeutic treatment and radiotherapy [31]. HIF-1*α* is the most critical hypoxia response molecular, and it significantly accumulates when cells are insulted with hypoxia [31]. The increasing level of HIF-1*α* was found in the prostate cancer tissues and was highly correlated with the metastatic risk of prostate cancer [32, 33]. TRPM7 was upregulated in the brain insulted with hypoxia and determined cell fate [17, 21]. Knockdown of TRPM7 reduced HIF-1*α* accumulation induced by TNF*α* [34], implying that TRPM7 likely serves a fundamental molecular accounting for the hypoxia response of cells. In this study, we found that hypoxia insult caused increased expression of TRPM7 simultaneously accompanied by the accumulation of HIF-1*α*. TRPM7 knockdown significantly reduced HIF-1*α* protein level without change of HIF-1*α* gene transcription in androgen-independent prostate cancer cells insulted with hypoxia. These findings, for the first time, demonstrate that TRPM7 exerts a crucial role in hypoxia-related signaling in androgen-independent prostate cancer cells, and suppression of TRPM7 promotes the degradation of HIF-1*α* under hypoxic conditions in androgen-independent prostate cancer cells.

Next, this study determined the underlying mechanisms involved in the regulation of HIF-1*α* degradation by TRPM7. The degradation of HIF-1*α* is regulated by the VHL-mediated oxygen-dependent signaling pathway and oxygen-independent signaling pathway involving the RACK1 and HSP90 molecular proteins [23]. Our study showed that suppression of TRPM7 increased the binding of RACK1 to HIF-1*α*, which could promote the degradation of HIF-1*α* [23]. Moreover, we also found that TRPM7 knockdown enhanced the phosphorylation of RACK1, which serves as the activated form to promote the binding ability of RACK1 [35]. These findings indicated that suppression of TRPM7 promoted the binding of RACK1 to HIF-1*α* through enhancing the phosphorylation of RACK1. In contrast with RACK1, the binding of HSP90 to HIF-1*α* stabilizes HIF-1*α* causing HIF-1*α* accumulation [36]. Moreover, RACK1 and HSP90 compete with each other for binding to HIF-1*α* to regulate its degradation [23]. It was further supported by our findings that when the binding of RACK1 to HIF-1*α* was increased by TRPM7 knockdown, the binding of HSP90 to HIF-1*α* was significantly reduced. Furthermore, we observed that knockdown of RACK1 led to the increase of interaction of HSP90 with HIF-1*α*. Our results further revealed that the regulation of HIF-1*α* degradation by TRPM7 knockdown was through the proteasome. In short, these results demonstrated that suppression of TRPM7 promoted HIF-1*α* degradation via strengthening the binding ability of RACK1 to HIF-1*α* through increased phosphorylation of RACK1. Moreover, RACK1 competes with HSP90 for the interaction with HIF-1*α* to regulate HIF-1*α* degradation. The mechanisms involved in the regulation of RACK1 phosphorylation by TRPM7 remain unknown. Ca^2+^ plays an essential role in the interaction between RACK1 and HIF-1*α*. Calcineurin, a calcium-/calmodulin-dependent and serine-/threonine-specific protein phosphatase, dephosphorylated RACK1 to promote HIF-1*α* accumulation [24]. TRPM7 mediated Ca^2+^ entry might activate the calcineurin phosphatase activity and subsequently to reduce the phosphorylation of RACK1 leading the accumulation of HIF-1*α*. Regarding the VHL-mediated oxygen-dependent signaling pathway, this study did not provide evidence if suppression of TRPM7 regulates the VHL-mediated oxygen-dependent signaling pathway accounting for the degradation of HIF-1*α*, which requires future investigation.

Next, we explored the downstream mediator of the TRPM7-HIF-1*α* signaling axis. Annexin A1 is a Ca^2+^ binding protein participating in vesicular trafficking as well as membrane organization, and thus to regulate cancer progression. Annexin A1 was upregulated in the leukemic cell line U937 exposed to hypoxia. The upregulation of Annexin A1 was inhibited or strengthened by HIF-1*α* knockdown or overexpression [8, 37]. It suggests that Annexin A1 is a downstream molecular of HIF-1*α*. The role of Annexin A1 in prostate cancer cells is under controversy. A decrease of Annexin A1 protein expression was observed in aggressive prostate cancer, and loss of Annexin A1 was associated with the tumorigenesis of prostate cancer [38, 39]. In contrast, hypoxia induced a significant increase of Annexin A1 protein expression and secretion, which promoted cell invasion and EMT [9]. Our finding is consistent with the latter. We observed that Annexin A1 protein expression was increased by hypoxia in androgen-independent prostate cancer cells, which was inhibited by knockdown of both HIF-1*α* and TRPM7. Furthermore, this study showed that knockdown of Annexin A1 significantly reduced EMT in androgen-independent prostate cancer cells under hypoxic conditions. Therefore, our findings reveal that Annexin A1 is a downstream molecular of TRPM7-HIF-1*α* signaling axis in prostate cancer under hypoxic conditions.

EMT is a process of onset of cancer cell migration and invasion that is polarized epithelial cells convert into nonpolarized, motile, and invasive mesenchymal cells [40]. Several molecular markers are indicating EMT. For example, the E-Cad, an epithelial cell-cell adhesion protein, is lost. In contrast, N-Cad as a mesenchymal cell-cell adhesion protein, as well as vimentin protein expression, are increased [41]. EMT is stimulated by various signals, including hypoxia, which was shown to prompted EMT in prostate cancer cells [22]. Here, our study also verified hypoxia insult caused a decrease of E-Cad protein expression, whereas an increase of N-Cad and vimentin protein expression. Reduction of HIF-1*α*, as well as downregulation of TRPM7, inhibited EMT and invasion of prostate cancer cells [15, 16, 42]. Our study is consistent with these findings, also showed that knockdown of both TRPM7 and HIF-1*α* inhibited EMT changes of androgen-independent prostate cancer cells induced by hypoxia, suggesting that TRPM7 regulates HIF-1*α*-mediated signaling to affect hypoxia-induced EMT in androgen-independent prostate cancer cells.

EMT stimulation leads to increased cell migration and invasion, which correlates with poor prognosis of prostate cancer [40, 43]. In line with other researcher's studies [9, 44], this study also showed that hypoxia significantly increased cell migration as well as invasion in androgen-independent prostate cancer cells. Moreover, we found that either HIF-1*α* or Annexin A1 knockdown significantly inhibited hypoxia-induced cell migration and invasion of androgen-independent prostate cancer cells, suggesting that HIF-1*α*/Annexin A1 signaling regulated by TRPM7 plays an essential role in the metastasis of prostate cancer under hypoxic condition.

In addition to Annexin A1, there are many other factors involved in EMT and invasion regulation by hypoxia and HIF-1*α*. For example, snail is one of the vital transcription factors that regulates EMT. It was stabilized by ubiquitin-specific protease 47 (USP47) which was regulated by transcription factor Sox9 in colorectal cancer cells under hypoxia [45]. Cysteine-rich protein 2 (CSRP2), as a novel target of HIF-1*α*, attributes to breast cancer cell invasion under hypoxic conditions by regulating the formation of invadopodium actin backbone [46]. It would be worthwhile determining in future experiments whether these factors also participate in EMT and invasion regulated by the TRPM7-HIF-1*α* signaling in prostate cancer under hypoxia.

**Figure 9:**
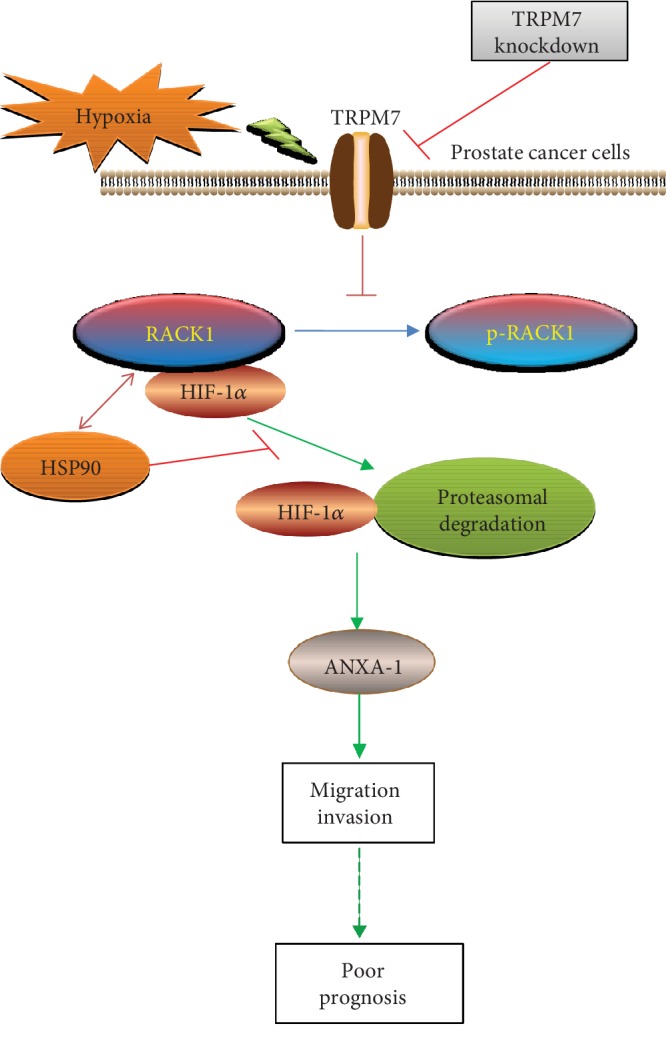
Schematic diagram of hypoxia-regulated TRPM7/HIF-1*α*/Annexin A1 signaling in androgen-independent prostate cancer cells. Hypoxia increased TRPM7 protein expression involving in the migration and invasion of androgen-independent prostate cancer cells, which implying poor prognosis. Suppression of TPRM7 inhibited dephosphorylation of RACK1 to enhance the binding of RACK1 to HIF-1*α* by competing with HSP90, then promoted the degradation of HIF-1*α* in the proteasome. Annexin A1 (ANXA1) acted as a downstream target for TRPM7/HIF-1*α* signaling controlling the migration and invasion of androgen-independent prostate cancer cells.

In summary, as shown in the schematic diagram (Figure 9), this study demonstrates that suppression of TRPM7 inhibits hypoxia-induced cell migration and invasion of androgen-independent prostate cancer cells by promoting the degradation of HIF-1*α*, with an underlying mechanisms involving the oxygen-independent RACK1-mediated HIF-1*α* degradation in the proteasome thought increased phosphorylation of RACK1. Moreover, suppression of TRPM7 abrogated HIF-1*α*/Annexin A1 signaling pathway to reduce hypoxia-induced EMT, cell migration, and invasion of androgen-independent prostate cancer cells. Our study further lightens the role of TRPM7 in the tumor biology of prostate cancer. It suggests the TRPM7-HIF-1*α*-Annexin A1 signaling pathway might serve as a potential drug target for the treatment of castrate-resistant prostate cancer.

## Data Availability

The data used to support the findings of this study are available from the corresponding author upon request.

## Supplementary Materials

Supplementary Materials Figure S1: HIF-1*α* protein expression of prostate cancer cells under normoxia (N) and hypoxia (H). PC3 cells (A) and DU145 cells (B) were exposed to hypoxia or normoxia for 24 hours. HIF-1*α* protein expression was determined by western blot. ∗ versus N group, *p* < 0.05, *n* = 4. Figure S2: co-IP of HIF-1*α* with RACK1 and HSP90 in PC3 prostate cancer cells under hypoxic condition. PC3 cells exposed to hypoxia for 24 h. Then cell lysates were incubated with either HIF-1*α* antibody or rabbit mAb IgG overnight using as negative control. Co-IP was carried out as indicated in Materials and Methods in the manuscript. Figure S3: TRPM7 knockdown restored the phosphorylation of RACK1 (p-RACK1), while enhanced the interaction between RACK1 and HIF-1*α* in DU145 cells. A, western blotting results showed the protein expression of p-RACK1, RACK1, and HSP90 in DU145 cells under normoxic (N), hypoxic condition (H), and hypoxia plus siRNA control (H + Si − Con) or siRNA-TRPM7 (H + Si − T7) for 24 h. ∗, # versus N and H + Si − Con, respectively, *p* < 0.05, *n* = 4. B, co-IP of HIF-1*α* with RACK1 and HSP90 after TRPM7 knockdown in DU145 cells under hypoxic condition. Figure S4: TRPM7 and RACK1 regulated HIF-1*α* degradation via the proteasome in DU145 cells under hypoxia. Cells with or without knockdown of TRPM7 (Si-T7) or overexpression of RACK1 (RACK1 group) were incubated with MG262 (1 *μ*M) for 6 h. HIF-1*α* protein expression was determined using western blot.
