# Supplementary material for: Suppression of TRPM7 Inhibited Hypoxia-Induced Migration and Invasion of Androgen-Independent Prostate Cancer Cells by Enhancing RACK1-Mediated Degradation of HIF-1α
Source: Oxid Med Cell Longev. 2020 Mar 6;2020:6724810. doi: 10.1155/2020/6724810 (PMC7079255; doi:10.1155/2020/6724810)
Supplement: Supplementary Materials — Figure S1: HIF-1α protein expression of prostate cancer cells under normoxia (N) and hypoxia (H). PC3 cells (A) and DU145 cells (B) were exposed to hypoxia or normoxia for 24 hours. HIF-1α protein expression was determined by western blot. ∗ versus N group, p < 0.05, n = 4. Figure S2: co-IP of HIF-1α with RACK1 and HSP90 in PC3 prostate cancer cells under hypoxic condition. PC3 cells exposed to hypoxia for 24 h. Then cell lysates were incubated with either HIF-1α antibody or rabbit mAb IgG overnight using as negative control. Co-IP was carried out as indicated in Materials and Methods in the manuscript. Figure S3: TRPM7 knockdown restored the phosphorylation of RACK1 (p-RACK1), while enhanced the interaction between RACK1 and HIF-1α in DU145 cells. A, western blotting results showed the protein expression of p-RACK1, RACK1, and HSP90 in DU145 cells under normoxic (N), hypoxic condition (H), and hypoxia plus siRNA control (H + Si − Con) or siRNA-TRPM7 (H + Si − T7) for 24 h. ∗, # versus N and H + Si − Con, respectively, p < 0.05, n = 4. B, co-IP of HIF-1α with RACK1 and HSP90 after TRPM7 knockdown in DU145 cells under hypoxic condition. Figure S4: TRPM7 and RACK1 regulated HIF-1α degradation via the proteasome in DU145 cells under hypoxia. Cells with or without knockdown of TRPM7 (Si-T7) or overexpression of RACK1 (RACK1 group) were incubated with MG262 (1 μM) for 6 h. HIF-1α protein expression was determined using western blot. [file 6724810.f1.docx]

**Supplementary Figures**

**Title: Suppression of TRPM7 Inhibited Hypoxia-induced Migration and Invasion of Androgen-independent Prostate Cancer Cells by Enhancing RACK1-mediated Degradation of HIF-1α**


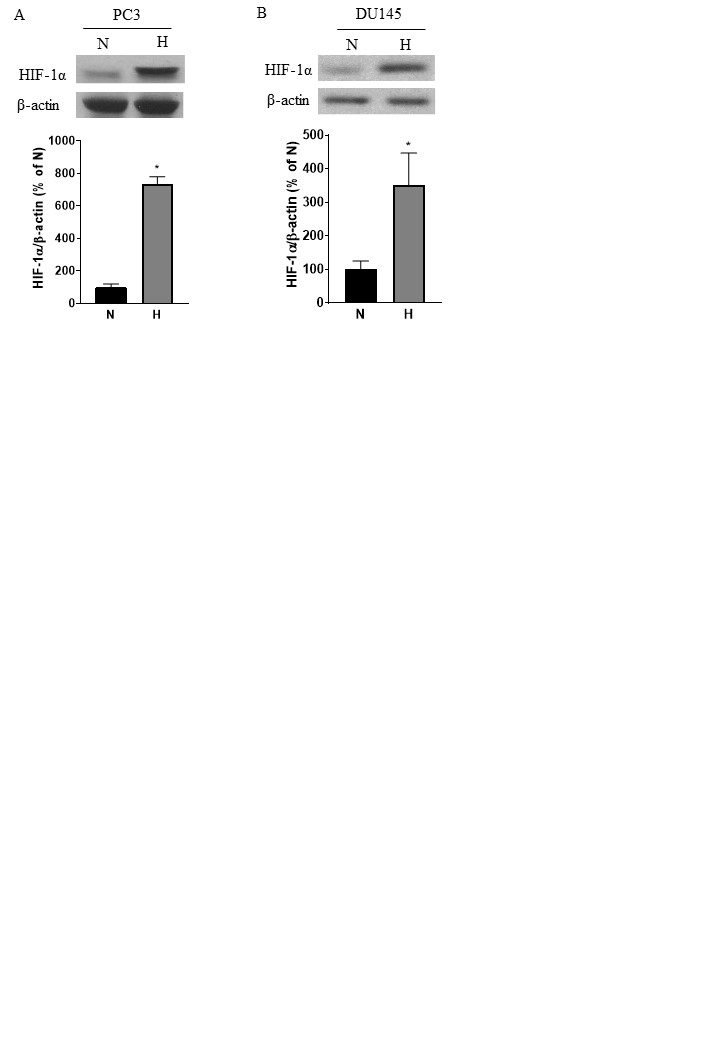


Figure S1. HIF-1α protein expression of prostate cancer cells under normoxia (N) and hypoxia (H). PC3 cells (A) and DU145 cells (B) were exposed to hypoxia or normoxia for 24 hours. HIF-1α protein expression was determined by western blot. * versus N group, p<0.05, n=4.
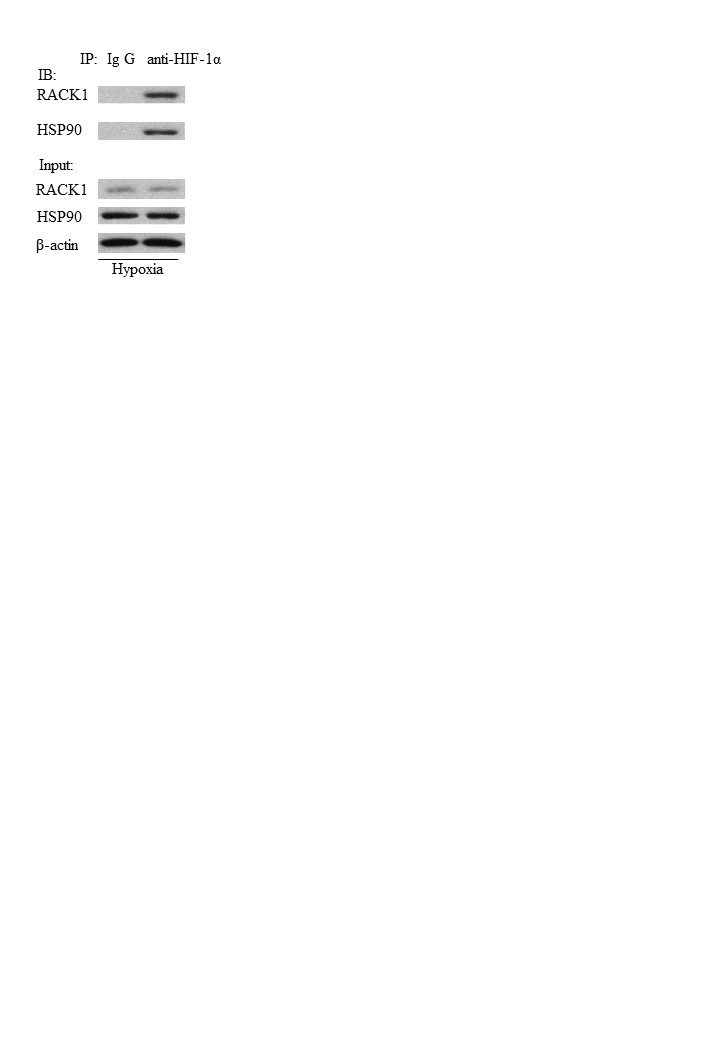


Figure S2. Co-IP of HIF-1α with RACK1 and HSP90 in PC3 prostate cancer cells under hypoxic condition. PC3 cells exposed to hypoxia for 24h. Then cell lysates were incubated with either HIF-1 α antibody or rabbit mAb Ig G overnight using as negative control. Co-IP was carried out as indicated in the Method section in manuscript.


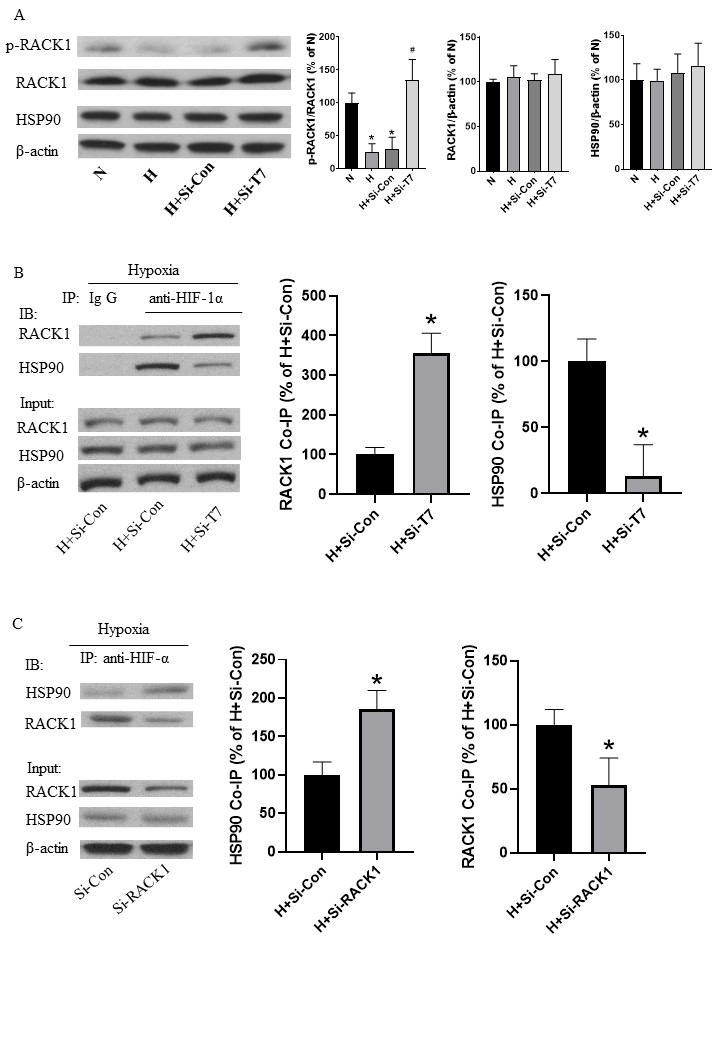


Figure S3. TRPM7 knockdown restored the phosphorylation of RACK1 (p-RACK1), while enhanced the interaction between RACK1 and HIF-1α in DU145 cells. A, western blotting results showed the protein expression of p-RACK1, RACK1 and HSP90 in DU145 cells under normoxic (N), hypoxic condition (H) and hypoxia plus siRNA control (H+Si-Con) or siRNA-TRPM7 (H+Si-T7) for 24h. *, # versus N and H+Si-Con, respectively, p<0.05, n=4. B, Co-IP of HIF-1α with RACK1 and HSP90 after TRPM7 knockdown in DU145 cells under hypoxic condition. * versus H+Si-Con, p<0.05, n=4. C, Co-IP of HIF-1α with RACK1 and HSP90 after RACK1 knockdown in DU145 cells under hypoxic condition. * versus H+Si-Con, p<0.05, n=4.


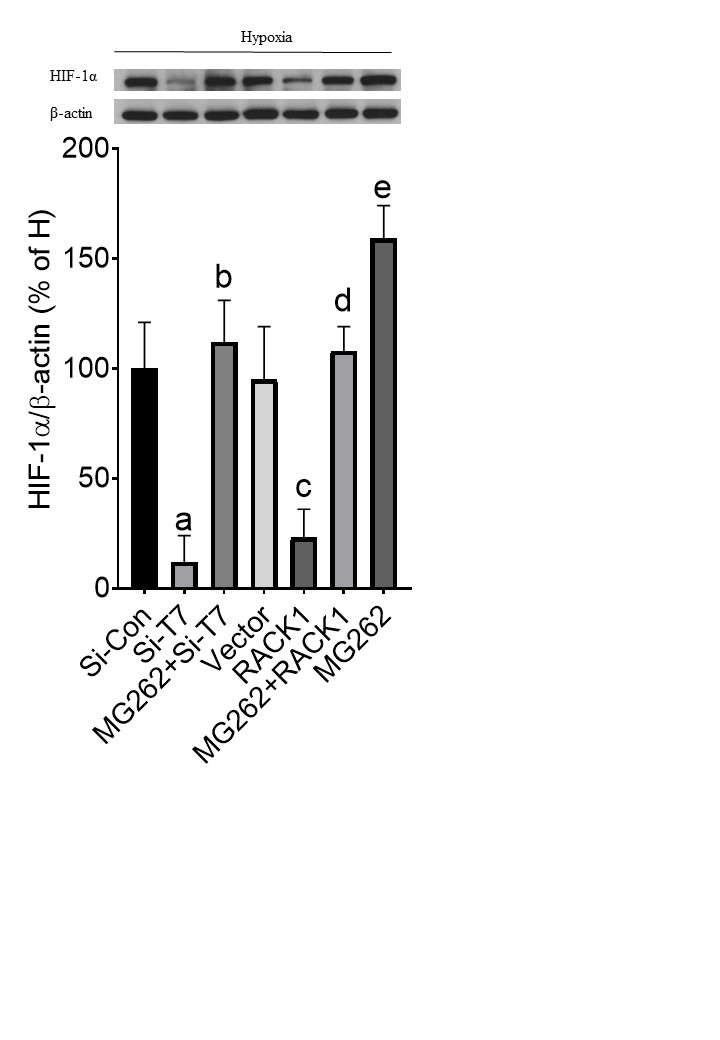


Figure S4. TRPM7 and RACK1 regulated HIF-1α degradation via the proteasome in DU145 cells under hypoxia. Cells with or without knockdown of TRPM7 (Si-T7) or overexpression of RACK1 (RACK1 group) were incubated with MG262 (1μM) for 6h. HIF-1α protein expression was determined using western blot. ^a^ versus Si-Con group, ^b^ versus Si-T7 group, ^c^ versus RACK1 group, ^d^ versus vector group, ^e^ versus Si-Con group, p<0.05, n=4.
